# GERONTOLOGY INTERNSHIPS, PRACTICUM, AND FIELD WORK FROM A FACULTY PERSPECTIVE

**DOI:** 10.1093/geroni/igad104.0488

**Published:** 2023-12-21

**Authors:** Rona Karasik

**Affiliations:** Saint Cloud State University, Saint Cloud, Minnesota, United States

## Abstract

While internships, practicum, and field work tend to be viewed primarily as student and site focused, academic faculty are also important to an internship’s success. Faculty roles may include assisting students in selecting and applying for site placements, ensuring that students are a “good fit” and properly prepared for a particular setting, overseeing quality control and assessment, guiding students to reflect on and harvest their learning throughout their internship, and at times, serving as advocate, trouble-shooter and/or mediator (Karasik, Donorfio, & Greenberg, 2023). In addition to student-facing responsibilities, faculty are also often involved in curricular aspects of internships and similar experiences (e.g., setting program parameters, expectations, and student learning goals and objectives), as well employing internship outcomes to academic program assessment (Karasik, 2009). Internship faculty also serve as a liaison between the academic program and community partners. Developing and maintaining community partnerships requires a broad skill-set, including but not limited to knowledge of the local aging network, collegiality, diplomacy, cultural and self-awareness, flexibility, and effective communication. Honing and consistently applying these traits is also essential, as recent studies suggest that at least some community partners perceive faculty involvement in community-based learning to be uneven and/or deficient (e.g., Karasik, 2020; Karasik & Hafner, 2021). Given the many roles faculty play in regard to internships and similar experiences, this presentation explores gerontology-based internships from a faculty perspective and shares the reflections and insight of one faculty member who has served as a gerontology internship coordinator for close to 30 years.

